# Indole-3-Carboxylic Acid From the Endophytic Fungus *Lasiodiplodia pseudotheobromae* LPS-1 as a Synergist Enhancing the Antagonism of Jasmonic Acid Against *Blumeria graminis* on Wheat

**DOI:** 10.3389/fcimb.2022.898500

**Published:** 2022-07-04

**Authors:** Yawei Que, Donghai Huang, Shuangjun Gong, Xuejiang Zhang, Bin Yuan, Minfeng Xue, Wenqi Shi, Fansong Zeng, Meilin Liu, Tingting Chen, Dazhao Yu, Xia Yan, Zhengyi Wang, Lijun Yang, Libo Xiang

**Affiliations:** ^1^ Key Laboratory of Integrated Pest Management of Crop in Central China, Ministry of Agriculture, Hubei Key Laboratory of Crop Diseases, Insect Pests and Weeds Control, Institute of Plant Protection and Soil Fertility, Hubei Academy of Agricultural Sciences, Wuhan, China; ^2^ Institute of Chinese Herbal Medicines, Hubei Academy of Agricultural Sciences, Enshi, China; ^3^ The Sainsbury Laboratory, Norwich Research Park, Norwich, United Kingdom; ^4^ State Key Laboratory for Rice Biology, Institute of Biotechnology, Zhejiang University, Hangzhou, China

**Keywords:** *Lsiodiplodia pseudotheobromae* LPS-1, indole-3-carboxylic acid, jasmonic acid, wheat powdery mildew, *Blumeria graminis*, synergist

## Abstract

The discovery of natural bioactive compounds from endophytes or medicinal plants against plant diseases is an attractive option for reducing the use of chemical fungicides. In this study, three compounds, indole-3-carbaldehyde, indole-3-carboxylic acid (3-ICA), and jasmonic acid (JA), were isolated from the EtOAc extract of the culture filtrate of the endophytic fungus *Lasiodiplodia pseudotheobromae* LPS-1, which was previously isolated from the medicinal plant, *Ilex cornuta*. Some experiments were conducted to further determine the antifungal activity of these compounds on wheat powdery mildew. The results showed that JA was much more bioactive than indole-3-carbaldehyde and 3-ICA against *Blumeria graminis*, and the disease severity caused by *B. graminis* decreased significantly with the concentration increase of JA treatment. The assay of the interaction of 3-ICA and JA indicated that there was a significant synergistic effect between the two compounds on *B. graminis* in each of the ratios of 3-ICA to JA (3-ICA:JA) ranging from 1:9 to 9:1. When the compound ratio of 3-ICA to JA was 2:8, the synergistic coefficient was the highest as 22.95. Meanwhile, a histological investigation indicated that, under the treatment of JA at 500 μg/ml or 3-ICA:JA (2:8) at 40 μg/ml, the appressorium development and haustorium formation of *B. graminis* were significantly inhibited. Taken together, we concluded that JA plays an important role in the infection process of *B. graminis* and that 3-ICA as a synergist of JA enhances the antagonism against wheat powdery mildew.

## Introduction

Powdery mildews (Ascomycota) encompass a category of widespread fungal pathogens that negatively impact a broad range of crops (Glawe, 2008). These fungi are obligate biotrophs, being completely dependent on living tissue for life, and are often host specific, associating with only one or a few species. For example, *Blumeria graminis* forma specialis *tritici* (*Bgt*) is known to be specific to wheat (*Triticum aestivum*) (Dean et al., 2012; Manser et al., 2021). Although *B. graminis* f. sp. *tritici*–resistant wheat cultivars have been developed, complete control of the disease has not been demonstrated and exogenous fungicide application is still necessary (Yang et al., 2008). Unfortunately, decades of application of agrochemical fungicides not only have produced pervasive environmental pollution but also have increased fungicide tolerance among fungal pathogen populations (Gong et al., 2013; Yang et al., 2013). An emerging alternative to the continued overuse of agrochemical fungicides is the adoption of naturally produced molecules, particularly those produced by plant endophytic microbes (Aly et al., 2010; Kharwar et al., 2011).

Endophytic microorganisms, both bacteria and fungi, carry out some part of their life cycle within living plant tissue and cause no disease symptoms (Tan and Zou, 2001; Rodriguez et al., 2009). Although most endophytes are of exogenous environmental original, some may be transmitted vertically in seeds or other generative tissue (Siegel et al., 1995; Rodriguez et al., 2009; Wei et al., 2014). Many endophytes form mutualistic relationships with their hosts, often considerably enhancing growth, defense, and adaptation to stress (Tan and Zou, 2001; Schardl et al., 2004; Rosenblueth and Martínez-Romero, 2006). In addition, endophytes may synthesize their own biologically active products, which can be applied as biocontrol agents either directly or indirectly, to stimulate induced resistance (Staniek et al., 2008; Aly et al., 2010; Kharwar et al., 2011; Vieira et al., 2014). Specifically, in some cases, the presence of endophytic microbes can produce amounts of bioactive secondary metabolites, including hormones, autocoids, and defense-related substances (Tudzynski, 1997; Pirttilä et al., 2004; Tanaka et al., 2005).

Endophytic microbes gained some notability in 1993 with the isolation of taxol, a multifunctional alkaloid, from the endophytic fungus *Taxomyces andreanae*, although endophytes have been mentioned in the literature since at least 1904 (Stierle et al., 1993; Qian et al., 2014). Since then, several other important compounds have been discovered to be produced by endophytic microbes, including isocoumarin (Findlay et al., 1995), camptothecin (Puri et al., 2005), podophyllotoxin (Eyberger et al., 2006), cochlioquinone A and isocochlioquinone A (Campos et al., 2008), and others (Schulz et al., 2002; Aly et al., 2010; Kharwar et al., 2011; Alvin et al., 2014). In *Lasiodiplodia pseudotheobromae* F2, isolated from *Illigera rhodantha* (Hernandiaceae) flower, six sulfureous diketopiperazines, lasiodiplines A–F, were characterized, and lasiodipline E was a potent antibacterial compound against the clinical strains *Streptococcus* sp., *Peptostreptococcus* sp., *Bacteroides vulgates*, and *Veillonella parvula*, respectively (Wei et al., 2014). In *L. pseudotheobromae* #1048 AMSTITYEL, isolated from stem of *Aegle marmelos*, two new compounds, lasdiplactone and lasdiploic acid, were isolated, which showed high xanthine oxidase inhibitory activity. In addition, the endophytic fungus #1048 AMSTITYEL showed maximum *in vitro* proteolytic and fibrinolytic activity (Meshram and Saxena, 2016; Kumar et al., 2019). In *L. pseudotheobromae* FKI-4499, isolated from soil collected in Okinawa Prefecture, Japan, aldsulfin, an anti-mannheimiosis agent, was identified, which displayed antibacterial activity against *Mannheimia haemolytica* and *Pasteurella multocida* (Sakai et al., 2021). In *L. pseudotheobromae* IBRL OS-64, isolated from the leaf of *Ocimum sanctum*, the fungal extract displayed significant antibacterial and anti-biofilm activities against methicillin-resistant *Staphylococcus aureus* (MRSA) and could be a candidate for antibacterial and antibiofilm drugs (Jalil and Ibrahim, 2021). In *L. pseudotheobromae* C1136, isolated from *Tridax procumbens* leaves, the active metabolites produced by the bioherbicidal isolates have bioherbicidal activity against *Amaranthus hybridus* L. and *Echinochloa crus-galli* weeds, and rhamnolipid, a biosurfactant produced by the bacterial *Pseudomonas aeruginosa* C1501, can serve as an adjuvant to improve the penetrability of bioherbicide active components from C1136 for controlling weeds (Adetunji and Oloke, 2013; Adetunji et al., 2017; Adetunji et al., 2018; Adetunji et al., 2020). However, the endophytic *L. pseudotheobromae* species have not been used in controlling agricultural diseases. Although natural products produced by endophytes are biodegradable, have low environmental toxicity, and show broad-spectrum bioactivity, these microorganisms remain an underutilized resource (Gunatilaka, 2006; Suryanarayanan et al., 2012). Such a class of safe, effective, and environment-friendly biocontrol agents would be particularly well suited for modern integrated management programs, aimed at reducing agrochemical use (Balandrin et al., 1985; Yang et al., 2008).

In our previous work, we isolated the endophytic fungus *L. pseudotheobromae* LPS-1 from the medicinal plant *Ilex cornuta* and found that the culture filtrate of the fungal isolate controlled *Bgt* infection more effectively than the broad-spectrum fungicide triadimefon (10 μg/ml) (Xiang et al., 2016). In this study, we sought to isolate and identify the specific secondary metabolites produced by *L. pseudotheobromae* LPS-1 that are antagonistic toward *B. graminis* on wheat. Here, we report the isolation and identification of three such compounds: indole-3-carbaldehyde (A2-5-6-1), indole-3-carboxylic acid (3-ICA; A2-5-6-2), and jasmonic acid (JA; A2-5-6-3). We further demonstrated that JA is antagonistic to appressorium development and haustorium formation during the *B. graminis* infection process, and 3-ICA synergistically enhances the antagonism of JA against *B. graminis* on wheat.

## Materials and Methods

### Plant Preparation, Fungal Strains, and Culture Conditions

Seeds of the powdery mildew disease-susceptible wheat variety “Zheng 98” were planted in 20-cm-diameter plastic pots, at a density of 10 plants per pot, and grew the seedlings out in a growth chamber for 10 days at 70% relative humidity, 18 ± 1°C, and under constant light (72 μmol m^−2^ s^−1^) conditions.

The *B. graminis* isolate E21 was obtained from Yilin Zhou of the Institute of Plant Protection (IPP), Chinese Academy of Agricultural Sciences (CAAS). Prior to inoculation of wheat plants, conidia were induced on excised segments of wheat leaf in a growth chamber for 10 days at 18 ± 1°C and under constant light (72 μmol m^−2^ s^−1^) conditions. *L. pseudotheobromae* LPS-1 was previously isolated from the medicinal plant, *Ilex cornuta*, at the Wuhan Botanical Garden in China. The internal transcribed spacer (ITS) and translation elongation factor 1α (TEF-1α) sequences were uploaded to GenBank under accession numbers KU180477 and KU180478, respectively (Xiang et al., 2016). LPS-1 was subcultured on potato dextrose agar (PDA) plates and then incubated at 25 ± 1°C for 3 days.

### Collection of LPS-1 Culture Filtrate

To collect the bioactive compounds produced by LPS-1, 300 ml of pre-sterilized potato dextrose broth (PDB) was poured into a 1-L Erlenmeyer flask and was aseptically inoculated with three 6-mm LPS-1 mycelial plugs and statically incubated at 25 ± 1°C for 7 days. When incubation was finished, the mycelial material was removed from the broth by Whatman paper filtration (Z240567, Sigma Aldrich, USA). The remaining broth was centrifuged for 10 min at 12,000 rpm. A total of 120 L of culture filtrate was collected for analysis.

### Compound Extraction and Isolation

The culture filtrate (120 L) was evaporated to extractum under reduced pressure at 50°C and extracted with petroleum ether and ethyl acetate (EtOAc) successively. A total of 35.7 g of petroleum ether extract and 197.2 g of EtOAc extract were obtained. The bioactivity of the aqueous phase and the above two extracts against *Bgt* E21 was tested, and it was found that the EtOAc extract had the highest biological activity and was thus chosen for further isolation and purification. The EtOAc extract (197.2 g) was dissolved in EtOAc, mixed with 100 mesh silica gel, and loaded onto a silica gel (5 cm × 10 cm, 300–400 mesh) chromatography column eluted with a gradient of petroleum ether/EtOAc (v/v: 50:1, 20:1, 10:1, 5:1, and 1:1) based on F_254_ silica gel thin-layer chromatography (TLC) monitoring. The same components were combined and concentrated to extractum under reduced pressure, and 10 fractions were obtained (A1~A10). The fraction A2, with the highest bioactivity against *Bgt* E21, was separated on thin silica gel H (5 cm × 30 cm) eluted with a gradient of petroleum ether/acetone first (v/v: 5:1 and 3:1) and then with a gradient of dichloromethane/methanol (CH_2_Cl_2_/MeOH) (v/v: 50:1, 30:1, 20:1, and 10:1) elution based on F_254_ silica gel TLC monitoring, obtaining 11 subfractions (A2-1~A2-11). The subfraction A2-5, with the highest bioactivity against *Bgt* E21, was further isolated by Sephadex LH-20 column (5 cm × 100 cm) eluted with MeOH based on F_254_ silica gel TLC monitoring, obtaining 12 subfractions (A2-5-1~A2-5-12). The subfraction A2-5-6, with the highest bioactivity against *Bgt* E21, was purified by high-performance liquid chromatography (HPLC) using a C_18_ column (2 cm × 25 cm; YMC Co., Ltd., Japan), yielding three compounds: A2-5-6-1 (19.2 mg), A2-5-6-2 (13.3 mg), and A2-5-6-3 (18.1 mg). A MeOH-H_2_O mixture was used as the mobile phase, and the flow rate was 10 ml/min. The UV detector was set to 220 nm. Purified compounds were identified as indole-3-carbaldehyde (A2-5-6-1), 3-ICA (A2-5-6-2), and JA (A2-5-6-3) using mass spectrometry (MS) and ^1^H and ^13^C nuclear magnetic resonance (NMR) (**Table 1**).

**Table 1:** ^1^H (150 MHz, CDCl_3_) and ^13^C NMR (150 MHz, CDCl_3_) spectroscopic data of A2-5-6-1, A2-5-6-2, and A2-5-6-3.

Position	A2-5-6-1		A2-5-6-2		A2-5-6-3	
	δ_H_	δ_C_	δ_H_	δ_C_	δ_H_	δ_C_
1	–	–	–	–	–	219.1
2	8.09 (s)	139.7	7.96 (s)	133.4	1.92 (1H, m)	54.0
3	–	120.2	–	120.2	2.13 (1H, m)	37.9
4	-	125.7	-	138.8	2.33, 1.52	27.3
5	–	138.9	–	127.7	2.78, 2.37	38.9
6	8.17 (d, J=8.1Hz)	122.4	8.08 (d, J=8.1Hz)	122.1	2.38 (2H)	25.6
7	7.24 (t, J=8.0Hz)	123.6	7.18 (t, J=8.1Hz)	122.4	5.26 (1H, m, J=18.3, 7.6)	124.9
8	7.28 (t, J=7.8Hz)	125.0	7.20 (t, J=7.8Hz)	123.6	5.47 (1H, m, J=18.1, 7.3)	134.4
9	7.48 (d, J=7.8Hz)	113.2	7.44 (d, J=7.8Hz)	112.9	2.06 (2H, m, J=7.3)	20.7
10/-CHO/ -COOH	11.08 (s)	187.4	-	169.2	0.96 (3H, t, J=7.5)	14.2
11	–	–	–	–	2.31 (2H)	37.9
12/-COOH	-	-	-	-	-	178.2

### Histological Investigation and Disease Severity Determination

To test their ability to control powdery mildew disease, the 3-ICA solution, the JA solution, and the 2:8 combination of 3-ICA:JA were applied to the wheat plants at the two-leaf stage with an automatic sprayer (PDE0012, Burkard Scientific Ltd., UK), with a spray volume of 350 ml/m^2^ and a pressure of 0.25 MPa. Treated wheat plants were air dried for 8 h and then inoculated with *Bgt* E21 at a density of 2 to 4 × 10^3^ conidia/cm^2^. Inoculated plants were incubated in a growth chamber at 18 ± 1°C, with the first 12 h in total darkness and thereafter in constant light (72 μmol m^−2^ s^−1^) conditions. When the incubation period was complete, plants were divided into two sets, either for histological examination of the *Bgt* E21 infection process or for assessment of powdery mildew disease severity. All experiments were repeated three times and utilized a randomized design with 12 replications.

For histological examination, leaf segments (3 cm) were excised from the midsection of primary leaves and subsequently stained with alcoholic lactophenol trypan blue (Koch and Slusarenko, 1990). At 8, 24, and 48 h post-inoculation (hpi), leaf segments were examined at magnification of ×10, and both ungerminated conidia and germinated conidia with primary germ tubes (with either normal or deformed appressoria) were counted within 10 haphazardly chosen fields. Meanwhile, the number of primary or mature haustoria per *Bgt* E21 colony was randomly scored from 30 colonies. To assess the disease severity of wheat powdery mildew, the number of *Bgt* E21 colonies presented on each leaf was counted at 7 days post-inoculation (dpi). The formula is as follows: Disease severity (%) = (diseased leaf area)/(total leaf area) × 100.

### Statistics and Assessment of Synergism

Dose-response regression was done by SPSS 22.0. The half maximal effective concentrations (EC_50_) of 3-ICA, JA, and their combinations were calculated according to the method of Gisi et al. (1985). Briefly, the calculated theoretical EC_50_ (EC_50(th)_) = (a + b)/(a/EC_50(A)_ + b/EC_50(B)_), where A and B represent 3-ICA and JA, respectively, and a and b are the ratios of the two compounds in the combinations. To categorize the type of interaction between 3-ICA and JA, we utilized the following ratio: *R* = EC_50(th)_/EC_50(ob)_, where EC_50(ob)_ is the EC_50_ value calculated according to the observed data. An additive response is indicated by an *R-*value between 0.5 and 1.5, a synergistic response is indicated by an *R-*value of greater than 1.5, and an antagonistic response is indicated by an *R-*value of less than 0.5. Treatment means were compared using the Duncan’s multiple range test, with significance set at the *P* = 0.05 level.

## Results

### Isolation of the Indole-3-Carbaldehyde, Indole-3-Carboxylic Acid, and Jasmonic Acid From LPS-1

**Figure 1:**
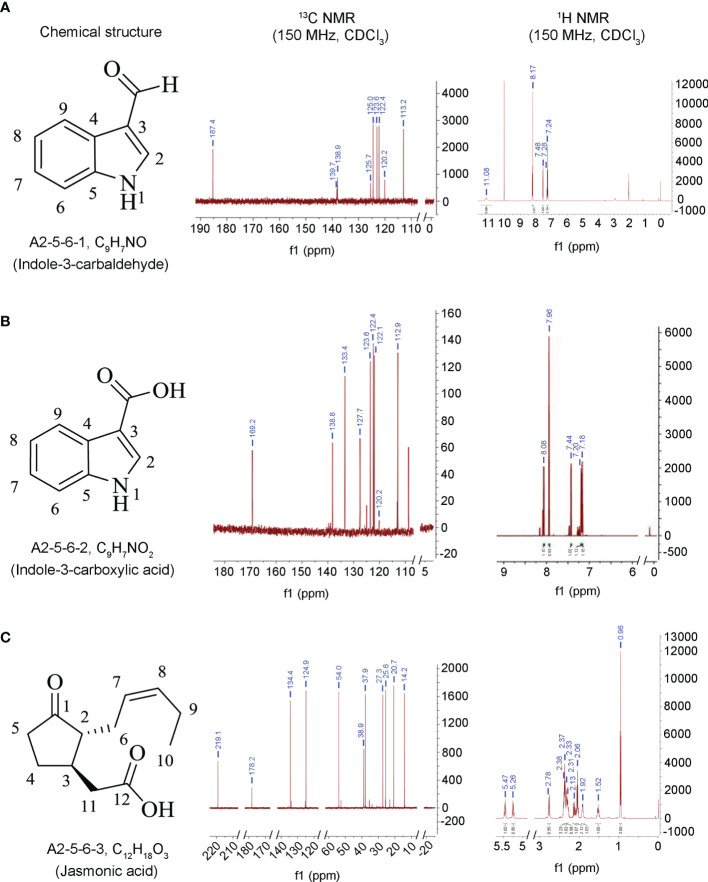
Chemical structure and NMR graphs of these compounds isolated from fermentation cultures of *L. pseudotheobromae* LPS-1. **(A)** A2-5-6-1, C_9_H_7_NO, indole-3-carbaldehyde. **(B)** A2-5-6-2, C_9_H_7_NO_2_, indole-3-carboxylic acid. **(C)** A2-5-6-3, C_12_H_18_O_3_, jasmonic acid.

Our previous work found that the culture filtrate of the endophytic fungus *L. pseudotheobromae* LPS-1 provided control of *Bgt* infection more effectively than the commercially available fungicide triadimefon (10 μg/ml) (Xiang et al., 2016). Therefore, to determine which secondary metabolites play a major role in antagonizing wheat powdery mildew, the compounds produced by the strain LPS-1 cultured in PDB broth for 7 days were isolated and identified. Three compounds (A2-5-6-1, A2-5-6-2, and A2-5-6-3) with relatively high concentrations were isolated from the ethyl acetate (EtOAc) extract using silica gel column chromatography as detailed in the experimental methods and were identified as indole-3-carbaldehyde (**Figure 1A**), 3-ICA (**Figure 1B**), and JA (**Figure 1C**). The MS, ^1^H NMR, and ^13^C NMR data (**Table 1**) were further compared to the literature, to confirm out results.

Compound A2-5-6-1 was obtained as white powder, and the molecular formula was determined as C_9_H_7_NO. ^1^H NMR (150 MHz, CDCl_3_): δ_H_ 8.09 (s), 8.17 (d, J = 8.1Hz), 7.24 (t, J = 8.0Hz), 7.28 (t, J = 7.8Hz), 7.48 (d, J = 7.8Hz), 11.08 (s). ^13^C NMR (150 MHz, CDCl_3_): δ_C_ 139.7, 120.2, 125.7, 138.9, 113.2, 122.4, 123.6, 125.0, 187.4 (**Table 1**). The spectral data were similar to that reported in the literature (Burton et al., 1986; Qian et al., 2014), and the compound was identified as indole-3-carbaldehyde (**Figure 1A**).

Compound A2-5-6-2 was also obtained as white powder, and the molecular formula was determined as C_9_H_7_NO_2_. ^1^H NMR (150 MHz, CDCl_3_): δ_H_ 7.96 (s), 8.08 (d, J = 8.1Hz), 7.18 (t, J = 8.1Hz), 7.20 (t, J=7.8Hz), 7.44 (d, J = 7.8Hz). ^13^C NMR (150 MHz, CDCl_3_): δ_C_ 133.4, 120.2, 127.7, 138.8, 112.9, 122.1, 122.4, 123.6, 169.2 (**Table 1**). The spectral data were similar to that reported in the literature (Burton et al., 1986; Qian et al., 2014), and the compound was identified as 3-ICA (**Figure 1B**).

Compound A2-5-6-3 was also obtained as white powder, and the molecular formula was determined as C_12_H_18_O_3_. ^1^H NMR (150 MHz, CDCl_3_): δ_H_ 1.92 (1H, m), 2.13 (1H, m), 2.33, 1.52, 2.78, 2.37, 2.38 (2H), 5.26 (1H, m, J = 18.3, 7.6), 5.47 (1H, m, J = 18.1, 7.3), 2.06 (2H, m, J = 7.3), 0.96 (3H, t, J = 7.5), 2.31 (2H). ^13^C NMR (150 MHz, CDCl_3_): δ_C_ 219.1, 54.0, 37.9, 27.3, 38.9, 25.6, 124.9, 134.4, 20.7, 14.2, 37.9, 178.2 (**Table 1**). The spectral data were similar to that reported in the literature (Husain et al., 1993), and the compound was identified as JA (**Figure 1C**).

### Disease Severity Determination

To further determine the antifungal activity of indole-3-carbaldehyde, 3-ICA, and JA against wheat powdery mildew, we tested the bioactivity of indole-3-carbaldehyde (with a concentration gradient of 2,500, 1,250, 625, 312.5, and 156.25 μg/ml), 3-ICA (with a concentration gradient of 2,500, 1,250, 625, 312.5, and 156.25 μg/ml), and JA (with a concentration gradient of 500, 250, 125, 62.5, and 31.25 μg/ml) against *Bgt* E21, respectively. Interestingly, there was no significant difference found in either disease severity or inhibition rate between either the indole-3-carbaldehyde or the 3-ICA treatments and the untreated control group (Mock). However, JA was much more bioactive than indole-3-carbaldehyde and 3-ICA against *Bgt* E21, and the disease severity decreased significantly, and inhibition rate increased significantly, with increasing JA concentration. When JA at 500 μg/ml was applied, the disease severity was 2.67 ± 0.53% and the inhibition rate was 96.95 ± 0.66% (**Table 2**), indicating that JA plays an important role in the infection process of wheat powdery mildew.

**Table 2:** The activities of indole-3-carbaldehyde, indole-3-carboxylic acid, and jasmonic acid against wheat powdery mildew in the laboratory.

Compound	Disease severity (%)	Inhibition rate (%)
Mock	87.19 ± 1.11	/
Indole-3-carbaldehyde (2500 μg/ml)	82.29 ± 0.97	5.72 ± 1.13
Indole-3-carbaldehyde (1250 μg/ml)	85.63 ± 1.25	1.91 ± 0.88
Indole-3-carbaldehyde (625 μg/ml)	86.67 ± 1.39	0.72 ± 0.58
Indole-3-carbaldehyde (312.5 μg/ml)	86.88 ± 0.83	0.46 ± 1.48
Indole-3-carbaldehyde (156.25 μg/ml)	87.08 ± 1.81	0.25 ± 0.91
Indole-3-carboxylic acid (2500 μg/ml)	79.38 ± 0.42	9.06 ± 1.09
Indole-3-carboxylic acid (1250 μg/ml)	82.71 ± 1.33	5.26 ± 0.34
Indole-3-carboxylic acid (625 μg/ml)	85.42 ± 1.11	2.15 ± 0.03
Indole-3-carboxylic acid (312.5 μg/ml)	87.08 ± 0.56	0.23 ± 0.89
Indole-3-carboxylic acid (156.25 μg/ml)	91.25 ± 0.83	-4.54±0.95
Jasmonic acid (500 μg/ml)	2.67 ± 0.53	96.95 ± 0.66
Jasmonic acid (250 μg/ml)	27.56 ± 0.75	68.41 ± 1.31
Jasmonic acid (125 μg/ml)	60.63 ± 0.83	30.55 ± 0.64
Jasmonic acid (62.5 μg/ml)	78.15 ± 1.63	10.48 ± 0.94
Jasmonic acid (31.25 μg/ml)	84.17 ± 0.28	3.57 ± 1.12

Disease severity (%) = (diseased leaf area)/(total leaf area) × 100. Inhibition rate (%) = (C − T)/C × 100, where C is the disease severity in the untreated control and T is the disease severity in the examined treatment.

**Figure 2:**
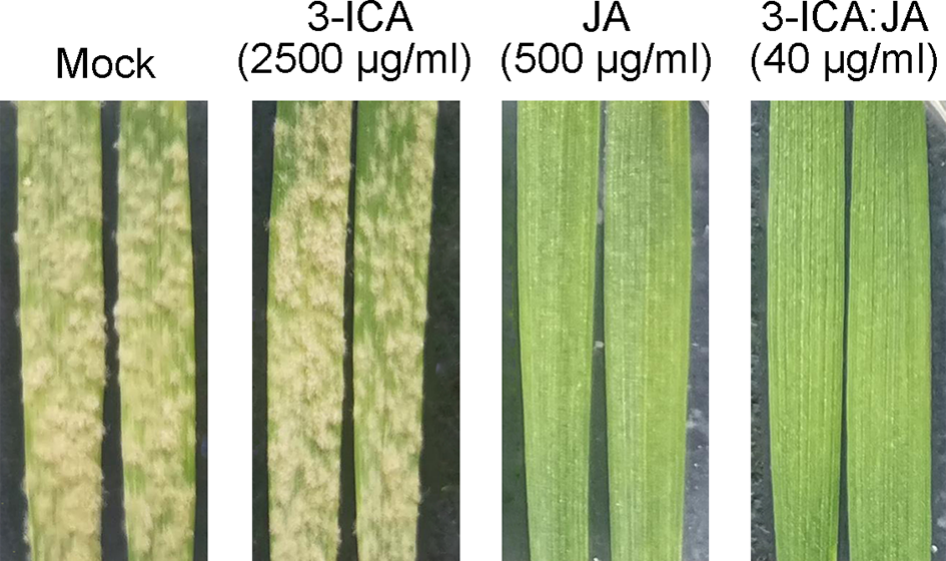
The activity of 3-ICA, JA, and their combinations against wheat powdery mildew at 2,500, 500, and 40 μg/ml, respectively. Mock, untreated control; 3-ICA, indole-3-carboxylic acid; JA, jasmonic acid; 3-ICA:JA, the 2:8 combination of indole-3-carboxylic acid and jasmonic acid.

Work by others has indicated that 3-ICA can be used as an antibiotic adjuvant of doxycycline toward a range of Gram-negative bacteria (Cadelis et al., 2021). Therefore, we wondered whether 3-ICA may interact with JA as an adjuvant to improve its bioactivity against wheat powdery mildew. We combined indole-3-carbaldehyde or 3-ICA with JA, and the compound ratio of indole-3-carbaldehyde in combination with JA (indole-3-carbaldehyde:JA) or 3-ICA in combination with JA (3-ICA:JA) was both 1:9, 2:8, 3:7, 4:6, 5:5, 6:4, 7:3, 8:2, and 9:1. Our results showed that the *R*-value (synergistic coefficient: theoretical EC_50_/observed EC_50_) of the combinations of 3-ICA and JA ranged from 2.1 to 22.95 for wheat powdery mildew. There was a synergistic effect when the combinations of the two compounds were at each ratio of 1:9-9:1 on this disease. Specifically, when 3-ICA and JA were combined in a ratio of 2:8, the EC_50_ was the lowest, at 9.05 μg/ml, and the synergistic coefficient was the highest, at 22.95 (**Table 3**). However, indole-3-carbaldehyde combined with JA did not produce significant synergism (**Table S1**). Meanwhile, we further tested the antagonism against *Bgt* E21 using the final concentration of 3-ICA at 2,500 μg/ml, JA at 500 μg/ml, and 3-ICA:JA (2:8) at 40 μg/ml, respectively. We found that *Bgt* E21 caused severe infection on susceptible wheat leaves in both the untreated control (Mock) and 3-ICA treatments. However, there were no visible disease symptoms observed on susceptible wheat leaves under the treatment of JA or 3-ICA:JA (2:8) (**Figure 2**). Overall, it appears that 3-ICA as a synergist of JA enhances the antagonism against wheat powdery mildew.

**Table 3:** The activities of indole-3-carboxylic acid, jasmonic acid, and their combinations on wheat powdery mildew in the laboratory.

Compound^*^	Regression equation	Observed EC_50_ (μg/ml) (95%Cl)	Theoretical EC_50_ (μg/ml)	Synergistic coefficient (*R*)
Indole-3-carboxylic acid	y=2.8374x-5.4965	17104.35 (6126.10-3119966.47)	/	/
Jasmonic acid	y=3.0195x-1.5743	166.57 (149.31-186.25)	/	/
Combination 1:9	y=1.6596x+3.4535	11.56 (10.09-13.21)	184.88	16.00
Combination 2:8	y=1.7592x+3.552	9.05 (7.84-10.39)	207.71	22.95
Combination 3:7	y=2.1868x+2.9831	10.81 (9.58-12.17)	236.97	21.92
Combination 4:6	y=2.6409x+1.0609	33.22 (29.96-36.96)	275.83	8.30
Combination 5:5	y=2.8643x+0.9787	25.51 (22.94-28.42)	329.93	12.93
Combination 6:4	y=1.808x+2.2082	35.74 (31.27-41.37)	410.44	11.48
Combination 7:3	y=1.3152x+2.815	44.00 (36.35-55.39)	542.91	12.34
Combination 8:2	y=2.7526x-1.0857	162.37 (143.10-185.69)	801.64	4.94
Combination 9:1	y=2.6571x-2.6941	730.80 (650.55-836.45)	1531.50	2.10

^*^The ratio of combination is indole-3-carboxylic acid to jasmonic acid.

### Histological Investigation of *B. graminis* Infection

**Figure 3:**
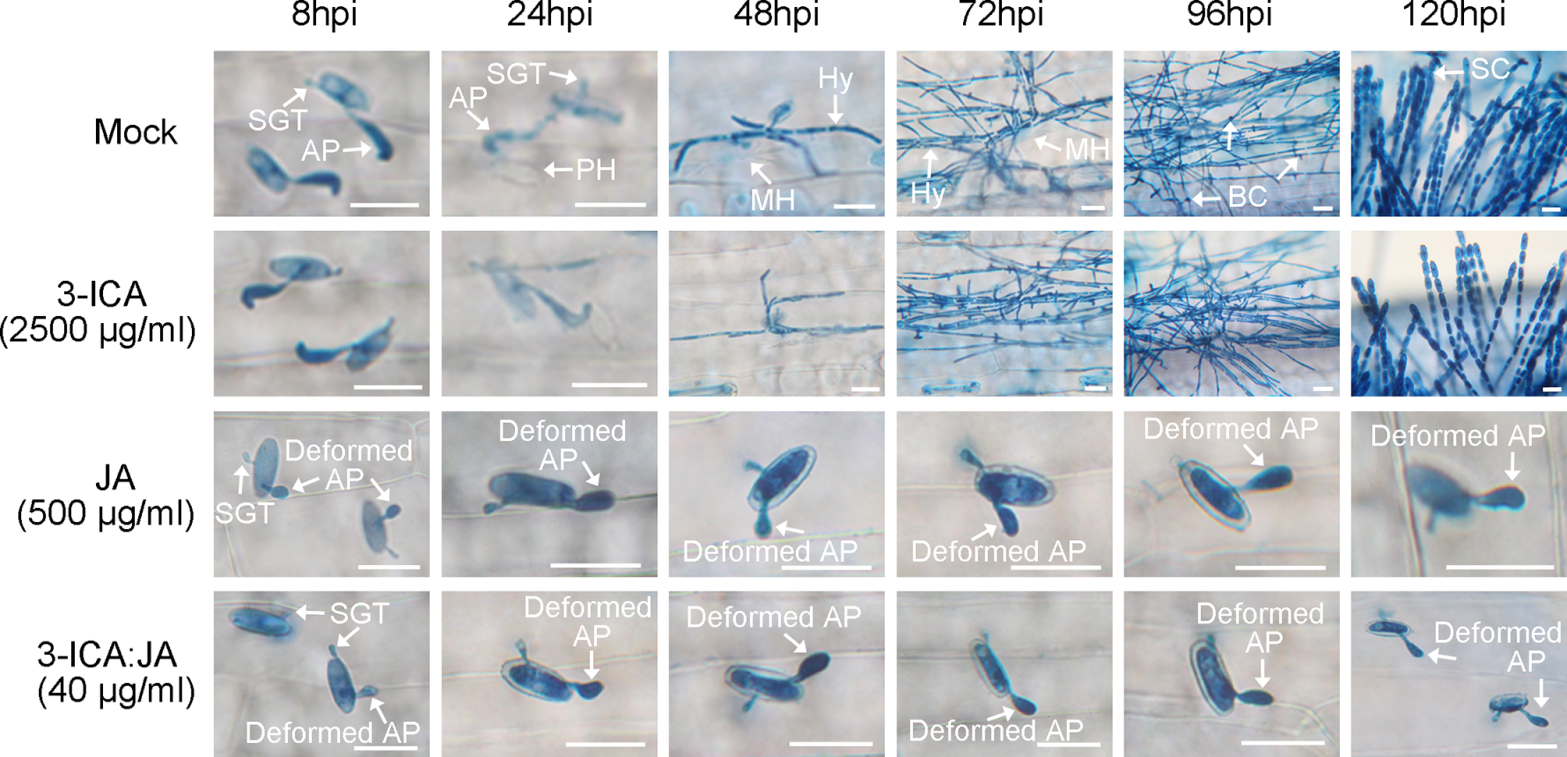
The development of *B graminis* on wheat leaves at different time courses in 3-ICA, JA, and their combination treatment. Mock, untreated control; 3-ICA, indole-3-carboxylic acid at 2,500 μg/ml; JA, jasmonic acid at 500 μg/ml; 3-ICA:JA, the 2:8 combination of indole-3-carboxylic acid and jasmonic acid at 40 μg/ml. Sampling time was 8, 24, 48, 72, 96, and 120 hpi, respectively. SGT, sticky germ tube; AP, appressorium; PH, primary haustorium; MH, mature haustorium; Hy, hypha; BC, base of conidiophores; SC, spore chain. The deformed appressoria failed to penetrate the host cells. Scale bar = 20 μm.

To determine the roles of 3-ICA and JA in the *Bgt* E21 infection process, we treated wheat leaves with either 3-ICA at 2,500 μg/ml, JA at 500 μg/ml, or 3-ICA : JA (2:8) at 40 μg/ml, 8 h prior to inoculation. The results showed that, in the untreated control (Mock) and 3-ICA treatment, wheat leaves were successfully infected and colonized by *Bgt* E21. By 8 hpi, appressoria were formed from germinated conidia. Primary haustoria were formed by 24 hpi and matured by 48 hpi, and then, secondary haustoria and hypha were formed by 72 hpi. The base of conidiophores emerged by 96 hpi and abundant fresh conidia reproduced by 120 hpi. However, in both the JA and 3-ICA:JA (2:8) treatments, the germinated conidia did not form normal appressoria, and the percentages of deformed appressoria were significantly higher (JA, 90.53 ± 0.91%; 3-ICA:JA, 89.68 ± 1.36%) than either the 3-ICA (5.85 ± 1.14%) or untreated control (6.79 ± 1.62%) treatments. Furthermore, the deformed appressoria failed to penetrate the host cells, and no haustoria were formed (**Figure 3**; **Table 4**). Meanwhile, at 24 hpi, conidia germinated at a rate of 83.47 ± 1.85% in the JA treatment, 83.44 ± 1.53% in the 3-ICA : JA treatment, 84.24 ± 1.65% in the 3-ICA treatment, and 86.43 ± 1.69% in the untreated control. In addition, at 24 hpi, appressoria were formed at a rate of 90.73 ± 0.78% in the JA treatment, 90.38 ± 1.85% in the 3-ICA:JA treatment, 91.22 ± 1.58% in the 3-ICA treatment, and 92.83 ± 0.60% in the untreated control. Overall, we found no significant difference in either the conidia germination rate or appressorium formation rate between the 3-ICA, JA, or 3-ICA : JA treatment and the untreated control (**Table 4**). These data suggest that JA plays important roles in appressorium development and haustorium formation against *B. graminis*.

**Table 4:** Histological investigation of the infection process of *B. graminis* after treatment with 3-ICA, JA, and their combinations.

Treatment	Conidium germination (%)	Appressorium formation (%)	The rate of deformed appressoria (%)	Primary haustorium formation (%)	Mature haustorium formation (%)
Mock	86.43 ± 1.69a	92.83 ± 0.60a	6.79 ± 1.62b	92.07 ± 0.94	99.06 ± 0.03
3-ICA (2500 μg/ml)	84.24 ± 1.65a	91.22 ± 1.58a	5.85 ± 1.14b	90.16 ± 1.06	98.76 ± 0.42
JA (500 μg/ml)	83.47 ± 1.85a	90.73 ± 0.78a	90.53 ± 0.91a	/	/
3-ICA:JA (2:8) (40 μg/ml)	83.44 ± 1.53a	90.38 ± 1.85a	89.68 ± 1.36a	/	/

3-ICA, indole-3-carboxylic acid at 2500 μg/ml; JA, jasmonic acid at 500 μg/ml; 3-ICA:JA (2:8), the 2:8 combination of indole-3-carboxylic acid and jasmonic acid at 40 μg/ml; /, no haustorium formation.

## Discussion

In this study, the endophytic fungus *L. pseudotheobromae* LPS-1 isolated from the medicinal plant, *Ilex cornuta*, can produce three agriculturally relevant bioactive compounds: indole-3-carbaldehyde, 3-ICA, and JA. Among them, both indole-3-carbaldehyde and 3-ICA are commonly present in microorganisms and plants and present antimicrobial and antitumor activities (Yue et al., 2000; Anderton et al., 2004; Kavitha et al., 2010; Mujahid et al., 2011; Qian et al., 2014; Liu et al., 2017; Zhou et al., 2019; Duong et al., 2021). Some studies have showed that indole-3-carbaldehyde, isolated from *Pseudomonas* sp. ST4, *Streptomyces* sp. CT37, *Aeromicrobium ponti* LGMB491, *Janthinobacterium lividum*, *Clitocybe nuda* LA82, *Angelica sinensis* callus, and *Marinomonas* sp., has inhibitory effects on the growth of *Sporisorium scitamineum*, *Ustilago maydis*, *Candida albicans* ATCC 10231, *Staphylococcus aureus*, and *Batrachochytrium dendrobatidis* JEL215, on zoospore germination of *Phytophthora capsici* PCM81, on neuroinflammation, and on the biofilm formation of *Vibrio cholerae* O1, respectively (Brucker et al., 2008; Chen et al., 2012; Rajalaxmi et al., 2016; Gos et al., 2017; Zhou et al., 2017; Liu et al., 2020; Fang et al., 2020). However, other reports about indole-3-carbaldehyde did not indicate its antimicrobial activity against pathogens. In this study, we also demonstrated that indole-3-carbaldehyde has no antifungal activity against *Bgt* E21 (**Table 2**). This indicates that indole-3-carbaldehyde may exhibit different extents of antimicrobial activity against different pathogens. Therefore, whether indole-3-carbaldehyde, isolated from *L. pseudotheobromae* LPS-1, has antimicrobial activity against other plant pathogens will be investigated in the future. For 3-ICA, in plants, it is regarded as an inactive auxin (IAA; indole-3-acetic acid) analog, and IAA is also implicated in plant defense (Bari and Jones, 2009; Borgati and Boaventura, 2011; Petti et al., 2012). Some studies indicate that 3-ICA, a plant cell wall–bound metabolite that could mediate accelerating callose accumulation in response to pathogens, may perform important functions as a mediator of induced resistance in plant basal defense against biotic stress. However, its function may be regulatory or signaling because it has no direct antifungal effect on pathogens (Forcat et al., 2010; Gamir et al., 2012; Iven et al., 2012; Gamir et al., 2014a; Gamir et al., 2014b; Frerigmann et al., 2016; Gamir et al., 2018; Pastor-Fernández et al., 2019). Similarly, in this study, we also found that there was no significant difference in disease severity and pathogenicity of *Bgt* E21 on susceptible wheat leaves between 3-ICA treatment and the untreated group (Mock) (**Figure 2**; **Table 2**), which preliminarily indicated that 3-ICA had no inhibitory effect on the infection process of wheat powdery mildew. Contrarily, JA was much more bioactive than indole-3-carbaldehyde and 3-ICA against *Bgt* E21, and the disease severity of *Bgt* E21 decreased significantly with the concentration increase of JA treatment (**Table 2**). In plants, the plant hormone JA and its derivatives are involved in regulating a diverse set of processes including cellular development, defense, and resistance to both abiotic and biotic stress (Ruan et al., 2019; Gomi, 2021). For instance, when applied to plants, the JA-derivative lasiojasmonate A (LasA), isolated from *Lasiodiplodia mediterranea*, a pathogen of grapes, induces many JA-regulated response *in planta* at both the genetic and physical level (Chini et al., 2018). The synthetic JA-Ile-macrolactone 5b acts to stimulate induced resistance against pests and pathogens in tea (Lin et al., 2020). In apple trees, JA has been shown to be more effective at stimulating induced resistance to *Tetranychus urticae* than the fungicide acibenzolar-S-methyl (BTH: benzothiadiazole) (Warabieda et al., 2020). Exogenous treatment with JA can enhance chilling tolerance of peach fruit and reduce the severity of internal flesh browning (Zhao et al., 2021). Some studies have also shown that JA signaling can induce the transcription of several defense-related transcription factors (TFs), such as MYC, MYB, NAC, ERF, and WRKY (Lorenzo et al., 2003; Delessert et al., 2005; Skibbe et al., 2008; Fernández-Calvo et al., 2011; Gao et al., 2011; Qi et al., 2011), and can also induce the MAP kinase cascade (Li et al., 2017), calcium channel activation (Kenton et al., 1999), and other processes that, along with other plant hormones like abscisic acid, salicylic acid, and ethylene, regulate plant growth, development, and stress response (Santner and Estelle, 2009; Ruan et al., 2019). JA is also present in other fungi in addition to *L. pseudotheobromae* LPS-1, such as *Acremonium* sp. D212, *Botryodiplodia theobromae*, *L. theobromae* strain 2334, and *L. iranensis* (Husain et al., 1993; Eng et al., 2016; Han et al., 2020; Shen et al., 2022). However, in this study, we do not know the molecular mechanism of 3-ICA and JA antagonizing *B. graminis* at present. Therefore, whether 3-ICA and JA served as signal molecules to induce the resistance of wheat against the infection of *B. graminis* will be further elucidated in the future.

Work by others has indicated that 3-ICA can be effectively employed as an antibiotic adjuvant of doxycycline against a range of Gram-negative bacteria (Cadelis et al., 2021). However, 3-ICA as a synergist of JA against plant pathogens has not been reported. Therefore, we wondered whether 3-ICA may interact with JA as an adjuvant to improve its bioactivity against fungal infection. We found that, in this study, there was indeed a synergistic effect between 3-ICA and JA against *Bgt* E21 on wheat in each of the ratios of 3-ICA to JA (3-ICA:JA) ranging from 1:9 to 9:1. When the compound ratio of 3-ICA to JA was 2:8, the synergistic coefficient was the highest as 22.95 (**Table 3**). Therefore, we chose the compound ratio of 3-ICA to JA as 2:8 for the following study. Similar to the treatment of JA at 500 μg/ml, there was no symptom on susceptible wheat leaves, and the appressorium and haustorium formation of *Bgt* E21 was significantly inhibited under the treatment of 3-ICA:JA (2:8) at 40 μg/ml (**Figures 2**, **3**; **Table 4**). This indicated that treatment with 3-ICA:JA (2:8) at 40 μg/ml was just as affective at controlling *Bgt* E21 infection as treatment with JA alone at 500 μg/ml, a 12.5× greater concentration of JA. It appears that 3-ICA significantly enhances the antagonistic efficiency of JA against wheat powdery mildew. In addition, to avoid the possibility of pathogenic resistance, antibiotic adjuvants should preferably lack their own antibiotic activity (Bernal et al., 2013), and we found that 3-ICA alone showed no direct activity against *Bgt* E21. Thus, 3-ICA can be employed as a synergist of JA to enhance the antagonism against *B. graminis*, and we suggest that the combination of JA and 3-ICA is potentially suitable for modern integrated disease management programs, aimed at reducing agrochemical use. These data further evince the untapped capacity of endophytic microorganisms to contribute useful compounds to agriculture and beyond.

## Data Availability Statement

The original contributions presented in the study are included in the article/**Supplementary Material**. Further inquiries can be directed to the corresponding authors.

## Author Contributions

Conceived and designed the experiments: YQ, LY, and LX. Performed the experiments: YQ, DH, SG, XZ, BY, MX, WS, FZ, ML, TC, DY, LY, and LX. Analyzed the data: YQ, LY and LX. Wrote the paper: YQ, XY, ZW, and LX.

## Funding

This work was supported by the Natural Science Foundation of China (Grant No. 31701834), the Natural Science Foundation of Hubei Province (Grant No. 2021CFB403), the China Agriculture Research System of MOF and MARA (Grant No. CARS-03), the Youth Science Foundation of Hubei Academy of Agricultural Sciences (Grant No. 2022NKYJJ08) and the Youth Science Foundation of Institute of Plant Protection and Soil Fertility, Hubei Academy of Agricultural Sciences (Grant No. 2021ZTSQJ13). The funders had no role in study design, data collection and analysis, decision to publish, or preparation of the manuscript.

## Conflict of Interest

The authors declare that the research was conducted in the absence of any commercial or financial relationships that could be construed as a potential conflict of interest.

## Publisher’s Note

All claims expressed in this article are solely those of the authors and do not necessarily represent those of their affiliated organizations, or those of the publisher, the editors and the reviewers. Any product that may be evaluated in this article, or claim that may be made by its manufacturer, is not guaranteed or endorsed by the publisher.

## Supplementary Material

Supplementary Table 1 The activities of indole-3-carbaldehyde, jasmonic acid, and their combinations on wheat powdery mildew in the laboratory.
