# Supplementary material for: Indole-3-Carboxylic Acid From the Endophytic Fungus Lasiodiplodia pseudotheobromae LPS-1 as a Synergist Enhancing the Antagonism of Jasmonic Acid Against Blumeria graminis on Wheat
Source: Front Cell Infect Microbiol. 2022 Jul 4;12:898500. doi: 10.3389/fcimb.2022.898500 (PMC9289256; doi:10.3389/fcimb.2022.898500)
Supplement: Supplementary Table 1 — The activities of indole-3-carbaldehyde, jasmonic acid, and their combinations on wheat powdery mildew in the laboratory. [file Table_1.docx]

Table S1 The activities of indole-3-carbaldehyde, jasmonic acid and their combinations on wheat powdery mildew in the laboratory.

| Compound^*^ | Regression equation | Observed EC_50_ (μg/ml) (95%Cl) | Theoretical EC_50_ (μg/ml) | Synergistic coefficient (*R*) |
| --- | --- | --- | --- | --- |
| Indole-3-carbaldehyde | y=1.1702x-0.4292 | 41586.14(8941.72-879136740.65) | / | / |
| Jasmonic acid | y=3.0109x-1.5511 | 166.05  (148.77-185.75) | / | / |
| Combination 1:9 | y=1.1293x+2.4903 | 175.98  (105.72-460.10) | 184.42 | 1.05 |
| Combination 2:8 | y=1.5719x+1.3853 | 183.61  (120.29-408.92) | 207.36 | 1.13 |
| Combination 3:7 | y=1.0426x+2.5693 | 208.64  (118.23-642.18) | 236.81 | 1.14 |
| Combination 4:6 | y=1.3856x+1.6813 | 273.41  (149.54-990.14) | 276.02 | 1.01 |
| Combination 5:5 | y=0.8493x+2.8211 | 346.94  (155.08-2321.54) | 330.78 | 0.95 |
| Combination 6:4 | y=1.2595x+1.4197 | 480.33  (199.52-7491.21) | 412.65 | 0.86 |
| Combination 7:3 | y=1.0366x+2.0571 | 781.32(251.55-26890.96) | 548.39 | 0.70 |
| Combination 8:2 | y=1.0507x+1.8407 | 854.01  (526.96-2084.06) | 817.20 | 0.96 |
| Combination 9:1 | y=1.1123x+1.5058 | 1657.92(621.03-8694722232.81) | 1602.90 | 0.97 |

^*^ The ratio of combination is indole-3-carbaldehyde to jasmonic acid.
